# Prevalence of hepatitis B virus infection in a cohort of HIV-positive women in Uganda: a cross-sectional survey at the "Zia Angelina" Medical Centre

**DOI:** 10.11604/pamj.2025.52.52.48853

**Published:** 2025-10-01

**Authors:** Francesco De Maria, Alice Joy Namuwonge, Fernando Rico González

**Affiliations:** 1 *Dipartimento di Scienze Mediche e Chirurgiche, Università Magna Graecia, Catanzaro, Italia*; 2 Zia Angelina Health Centre of Kampala, Kampala, Uganda

**Keywords:** Hepatitis B virus, HIV, women

## To the editors of the Pan African Medical Journal

Hepatitis B virus (HBV) infection remains a significant cause of morbidity and mortality worldwide, particularly in sub-Saharan Africa, where HIV-HBV coinfection is common [1]. Recent studies estimate that up to 20-30% of people living with HIV (PLHIV) in this region may be coinfected with HBV [2]. Despite updated WHO guidelines recommending universal HBsAg screening for PLWH [3], access to systematic screening and follow-up remains limited in many low-resource settings. We conducted a descriptive cross-sectional study at the “Zia Angelina” Medical Centre in Kampala, Uganda, between November 2021 and August 2022.

Inclusion criteria: all HIV-positive women aged ≥18 years attending routine clinical follow-up during the study period and providing written informed consent were eligible. Men and women under 18 years were excluded. Sampling strategy and rationale: a convenience sampling approach was used, consecutively enrolling all eligible women presenting during the study period. Although no formal sample size calculation was performed, the final cohort of 153 women was considered sufficient to provide preliminary estimates of HBV prevalence in this specific population. Participants were tested for HBsAg using a validated rapid assay. Among them, five (3.3%) were positive. All coinfected individuals showed normal aspartate aminotransferase (AST), Alanine transaminase (ALT), and platelet levels, with average FIB-4 scores of 1.80 (range 1.35-2.12), indicating no significant hepatic fibrosis. One patient was pregnant at the time of testing.

Although HBV DNA testing was unavailable, the absence of biochemical abnormalities suggests inactive or low-replication infection [4]. Our findings align with other African studies reporting HBV prevalence between 2% and 10% among HIV-positive individuals [1,2]. Importantly, the exclusive enrollment of women highlights considerations related to reproductive health, including prevention of mother-to-child transmission and the need for prophylaxis during pregnancy [5,6]. These results support the integration of HBV screening into HIV care, in line with WHO guidelines [3,7]. Even a small number of undiagnosed cases may lead to long-term complications, considering that chronic HBV affects an estimated 60-80 million individuals in sub-Saharan Africa [1]. The use of simple, non-invasive tools such as FIB-4 may provide a pragmatic approach to monitoring liver disease in low-resource contexts [4]. Our findings also align with previous work at the same facility on HCV prevalence among PLWH, suggesting the feasibility of integrated viral hepatitis screening [8]. Moreover, future studies should account for hepatitis D virus (HDV) coinfection, which complicates clinical management and worsens prognosis [9].

## Conclusion

This study highlights a low but clinically significant prevalence of HBV infection among HIV-positive women in Uganda, with no evidence of advanced liver fibrosis. Integration of HBV screening into HIV services is essential, particularly for women of reproductive age, to prevent long-term complications and move towards the WHO´s 2030 elimination goals [1,3,7,10].
